# Key factors to establish the ovalbumin-induced atopic dermatitis minipig model: age and body weight

**DOI:** 10.1186/s42826-022-00141-4

**Published:** 2022-10-20

**Authors:** Young Kyu Kim, JuKyung Lee, Hyeon-Young Kim, Sung-Hwan Kim, Jeong Ho Hwang, Han Na Suh

**Affiliations:** 1 Animal Model Research Group, Korea Institute of Toxicology, 30 Baekhak1-gil, 56212 Jeongeup, Jellabuk-do Republic of Korea; 2 Department of Medical IT Convergence, Kumoh National Institute of Technology, 39177 Gumi, Gyeongbuk Korea; 3 Human Health Risk Assessment Center, Korea Institute of Toxicology, 30 Baekhak1-gil, 56212 Jeongeup, Jellabuk-do Republic of Korea; 4 Center for Companion Animal New Drug Development, Korea Institute of Toxicology, 30 Baekhak1-gil, 56212 Jeongeup, Jellabuk-do Republic of Korea

**Keywords:** Yucatan minipig, Atopic dermatitis, Ovalbumin, Age/body weight, Dermal thickness

## Abstract

**Background:**

Given its similar structure and immune response to the human skin, porcine is a good model for dermal studies. Here, we sensitized ovalbumin (Ova) on minipig back skin for 2–4 weeks to induce chronic atopic dermatitis (AD).

**Results:**

Gross observation, serum cytokine level, epidermal thickness, and epidermal integrity did not change after 4 weeks of Ova induction compared with the control, indicating AD modeling failure. Only the neutrophils in the blood and macrophages in bronchoalveolar lavage fluid changed slightly until 3 or 2 weeks after Ova sensitization, respectively. The successful and failed Ova-induced AD minipig models only differ in age and body weight of the minipigs. The minipigs, 12 months old with a 30-kg median weight, had a two-fold thicker dermis than minipigs 8–10 months old, with an 18.97-kg median weight, resulting in impaired Ova permeability and immune response.

**Conclusion:**

Age and body weight are key factors that should be considered when developing an Ova-induced AD minipig model.

**Supplementary Information:**

The online version contains supplementary material available at 10.1186/s42826-022-00141-4.

## Background

The skin is an organ of the integumentary system comprising three layers: epidermis, dermis, and subcutaneous. The epidermis serves as a mechanical barrier between the outside and inside of the body, preventing the exogenous penetration and promoting water retention. The dermis contains connective tissue, blood vessels, apocrine/sebaceous glands, nerves, and hair follicles. The subcutaneous layer contains most body fat to insulate the muscles and internal organs. The skin is directly exposed to the outside environment; thus, its immune system is well developed. Langerhans cells, skin resident macrophages, are located in the stratum spinosum of the epidermis and maintain skin homeostasis [1]. Other types of immune cells, including dendritic cells, different types of macrophages, T lymphocytes, and natural killer cells, are mainly found in the dermis. Skin immunity depends on the dermal blood vessels and lymph nodes in the dermis because of the easy recruitment of immune cells during disease conditions.

Atopic dermatitis (AD) alters the skin immunity, leading to mechanical-barrier defects. Increased irritation due to loss of barrier function is associated with a severe immune response in the skin. Thus, treatment for AD primarily focuses on restoring skin barrier function, reducing inflammation, and alleviating symptoms. Generally, topical treatments, such as corticosteroids, calcineurin inhibitors, or antibiotics combined with oral antihistamine drugs, are applied [2]. A specific cytokine blocker (monoclonal antibody) is also used to treat the AD condition [3–5]. AD is chronic and relapses easily; hence, novel effective drugs are still being developed to overcome these problems. Moreover, reliable and human mimic animal models are essential in determining the effective drug dose without toxicity.

Rodents have been primarily used as an AD model due to their ease of handling and cost-effectiveness. Thus, methods for modeling AD in mice have been well-established, such as epicutaneous sensitization of house dust mites [6], *S.aureus* infection [7], food allergy [8], hapten induction [9], and genetically engineered mouse models [10, 11]. However, mouse skin differs from human or porcine skin in anatomical, physiological, and biochemical aspects. Rodents have loose subcutaneous connective tissues, while those of porcine and humans have a tight connection [12, 13]. Humans have eccrine and apocrine sweat glands, whereas porcine only has apocrine glands [14] and rodents have eccrine glands in their foot pads [15]. The epidermal thickness of humans is 50–120 μm, porcine is 30–140 μm, and rodents is 10–45 μm [13, 16]. Additionally, components of epidermal immune cells are Langerhans and αβ T cells in humans, whereas γδ dendritic epidermal T cells are dominant in mice [17–20]. Epidermal cells are renewed every 30 days for porcine, 26–28 days for humans, and 8–10 days for rodents [21].

The similar epidermal thickness and immunological reactivity [22] of porcine to those of humans result in comparable drug absorption and metabolism [23]. Those similarities make porcine a superior animal model for dermal disease studies and drug development. We previously developed a 2-week ovalbumin (Ova)-sensitized AD minipig model that is comparable with human AD, as evidenced by histological and cytokine analyses in serum and skin [24]. Considering that AD is a chronic disease, we hypothesized that prolonging the Ova sensitization mediates an accurate and precise AD minipig model. In this study, we sensitized Ova for 2, 3, or 4 weeks to optimize the AD minipig model and analyzed the histopathology and cytokine levels in serum.

## Results

### Establishment of chronic atopic dermatitis applying topical ovalbumin

We previously established a reliable AD minipig model [24]. However, a 2-week sensitization period is insufficient to reflect chronic AD. Thus, we extended the sensitization duration to 4 weeks. First, hematological analysis was performed to determine the immune response during AD induction. Compared with the control, the Ova-treated minipigs had higher neutrophil content until 3 weeks after sensitization.

**Fig. 1:**
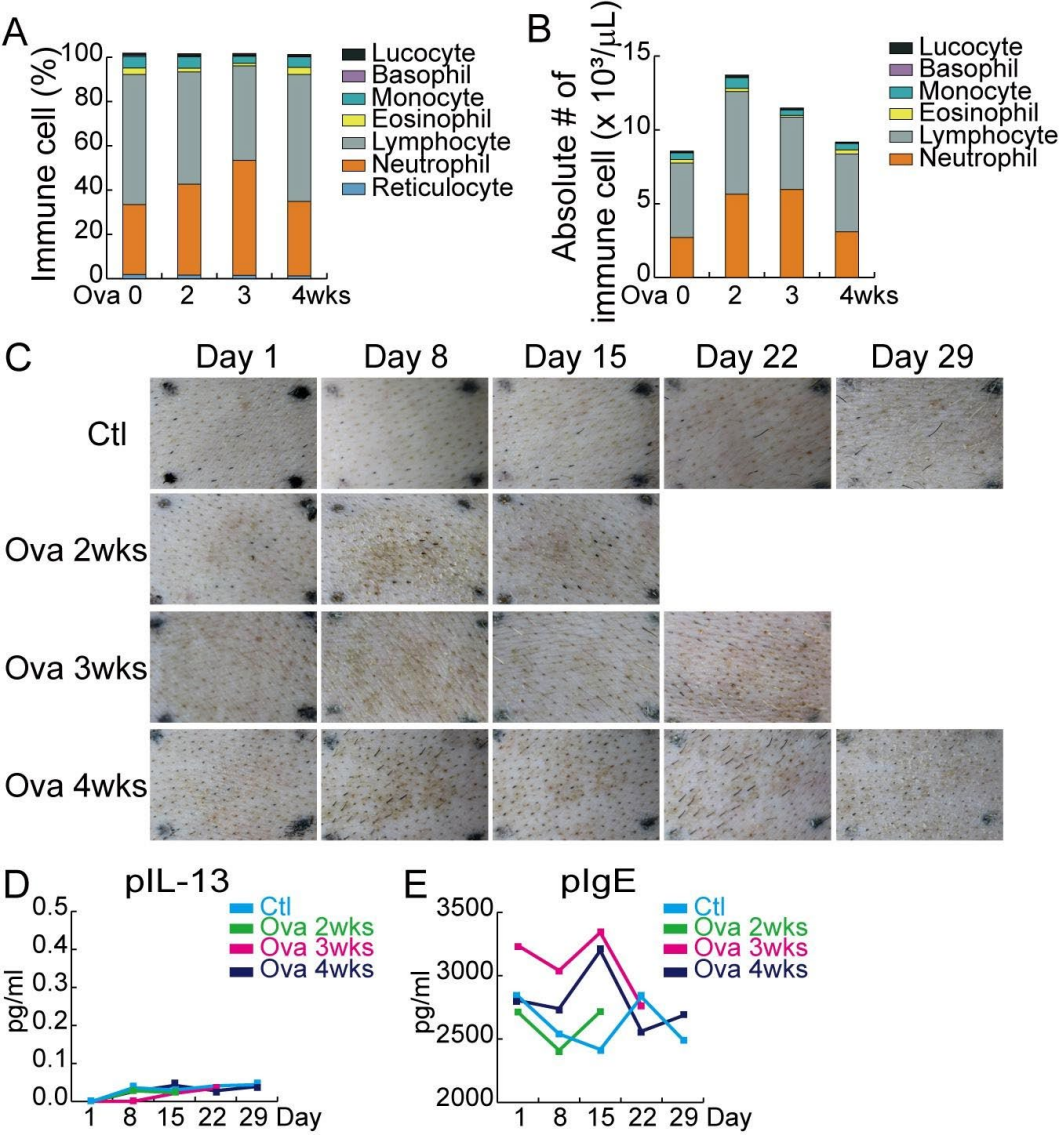
**Confirmation of AD** **(A–B)** Hematology was measured using an ADVIA2120i hematology analyzer (Siemens, USA). Relative **(A)** and absolute populations **(B)** are indicated. **(C**) Macroscopic analysis of Ova-induced AD shown in images of day 1, 8, 15, 22, and 29, which best represent the changes, selected from images of four sensitized skin sites from one minipig. **(D–E)** Analysis of the absolute cytokine protein level in serum at day 1, 8, 15, 22, and 29 of Ova sensitization as quantified by ELISA. **(D)** porcine (*p*) IL-13, **(E)** porcine IgE. Ctl: control; Ova: ovalbumin

Moreover, monocytes slightly increased until 2 weeks after sensitization (Fig. 1 A–1B), and gross observation and serum cytokine analysis were performed to confirm the AD modeling. Ova-treated skin sites did not show redness and hyperkeratosis after 4 weeks of sensitization (Fig. 1 C). Additionally, the levels of cytokine (IL-13) and immunoglobulin E did not change in serum after Ova sensitization (Fig. 1D and E). IL-4 and interferonγ were not detected during Ova induction (data not shown). These results indicate the failure of 4 weeks of Ova-induced AD minipig modeling.

## Difference in skin layer thickness and integrity depends on age and body weight

**Fig. 2:**
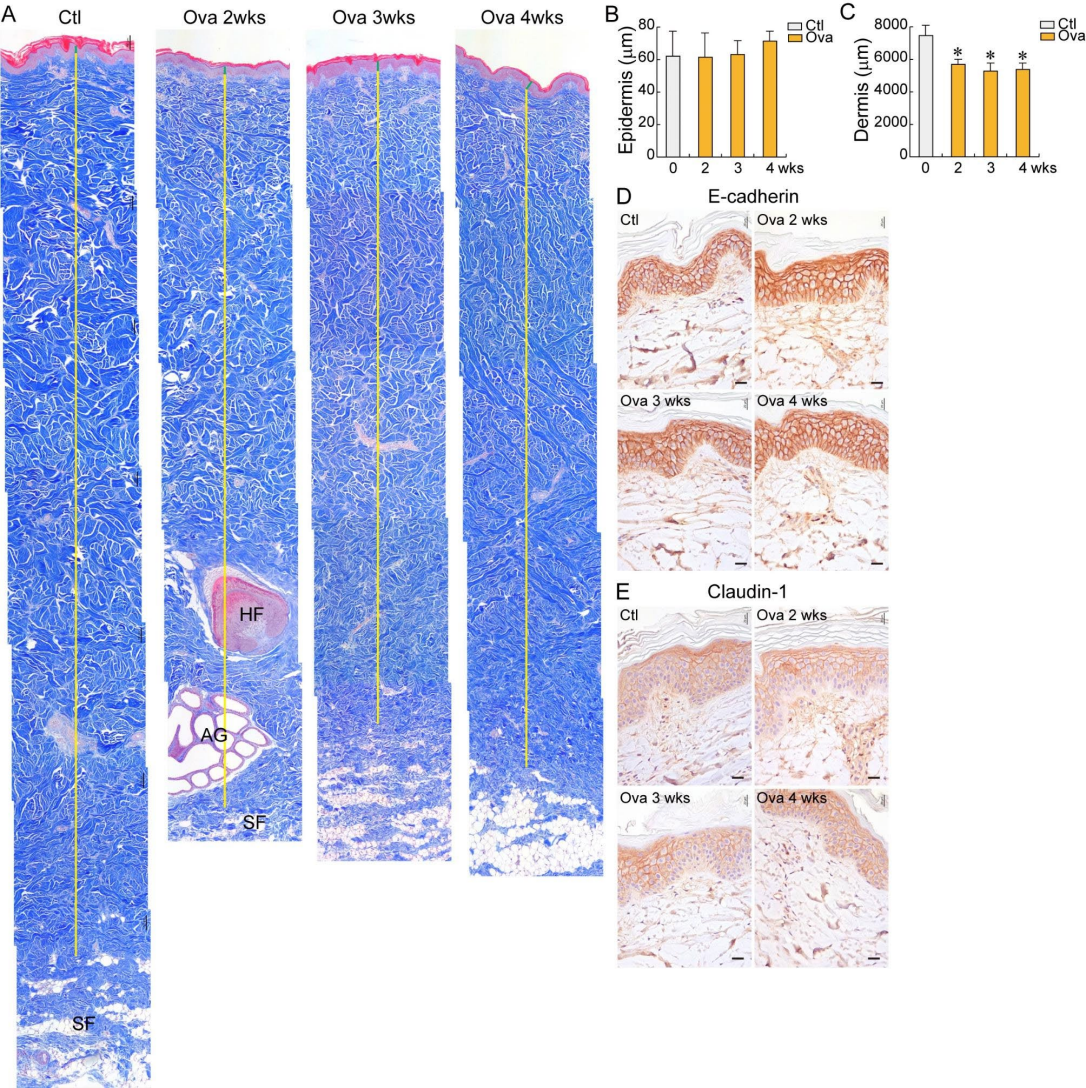
**Histological analysis of skin layers in minipigs aged 12 months with 30 kg median body weight** Minipigs were treated with Ova during 2, 3, 4 weeks. **(A–C)** Masson’s trichrome staining. **(A)** Histology of skin layers from the epidermis to the subcutaneous. Green line: epidermis; Yellow line: dermis; SF: subcutaneous fat; HF: hair follicle; AG: apocrine gland. **(B)** Thickness of the epidermis. **(C)** Thickness of the dermis. p* < 0.05. **(D-E)** Immunohistochemistry. **(D)** E-cadherin; **(E)** Claudin-1. Scale bar = 100 μm; Ctl: control; Ova: ovalbumin

Although the same method was used to develop the Ova-induced AD minipig model, there was still a discrepancy between the models in our previous and current studies. In this study, we utilized Yucatan minipigs 12 months old and weighing 25.37–30.74 kg (median weight: 30 kg). The control and Ova-treated skin samples showed no difference in epidermal thickness (Fig. 2 A–2B), but the Ova-treated skin had a thinner dermal layer than the nontreated skin (Fig. 2 A, 2 C) after 2 weeks of Ova induction. Also, epithelial integrity did not change after 4 weeks of the Ova induction, as identified by cell–cell junction protein levels (E-cadherin: adherent junction; Claudin-1: tight junction) (Fig. 2D and E).

**Fig. 3:**
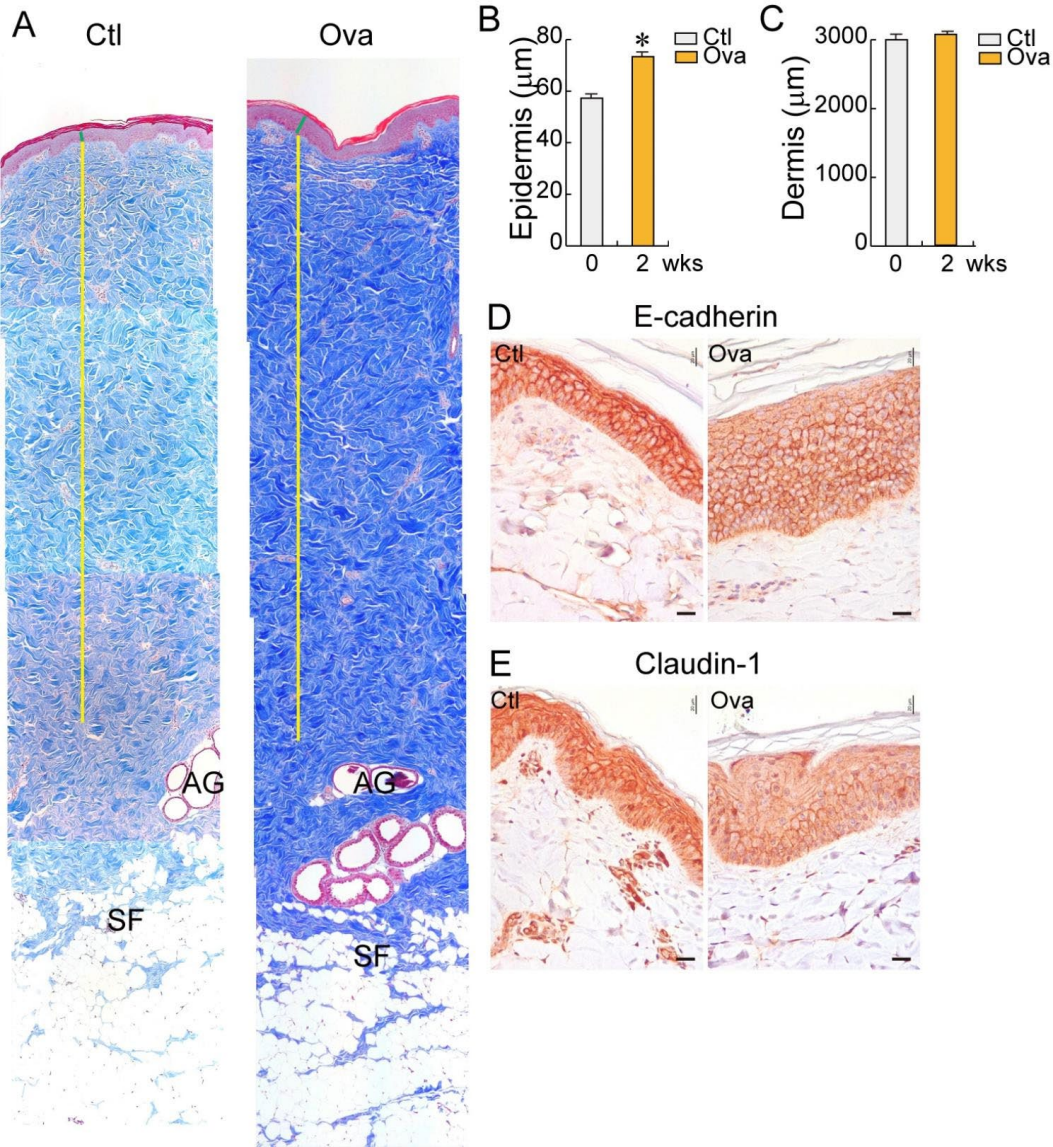
**Histological analysis of skin layers in minipigs aged 8–10 months with 18.97 kg median body weight** Minipigs were treated with Ova during 2 weeks. **(A–C)** Masson’s trichrome staining. **(A)** Histology of skin layers from the epidermis to the subcutaneous. Green line: epidermis; Yellow line: dermis; SF: subcutaneous fat; AG: apocrine gland. **(B)** Thickness of the epidermis. **(C)** Thickness of the dermis. p* < 0.05 **(D–E)** Immunohistochemistry. **(D)** E-cadherin; **(E)** Claudin-1. Scale bar = 100 μm; Ctl: control; Ova: ovalbumin

The Ova induction to the Yucatan minipigs 8–10 months old and weighing 15.05–21.17 kg (median weight: 18.97 kg) increased the epidermal thickness but did not affect dermal thickness (Fig. 3 A–3 C). The protein expression levels of E-cadherin and claudin-1 decreased in the epithelium after 2-weeks Ova sensitization, indicating that the epithelial integrity decreased (Fig. 3D and E). The dermal thickness notably increased (≒ 3000 μm vs. 6000 μm; 50% increase) in the minipigs with different ages (9 vs. 12 months) and body weight (18.97 vs. 30 kg) (Fig. 2 C, 3 C). These findings suggest that age, weight, and related dermal thickness play crucial roles in the establishment of an AD minipig model.

## Correlation between local and systemic inflammations

**Fig. 4:**
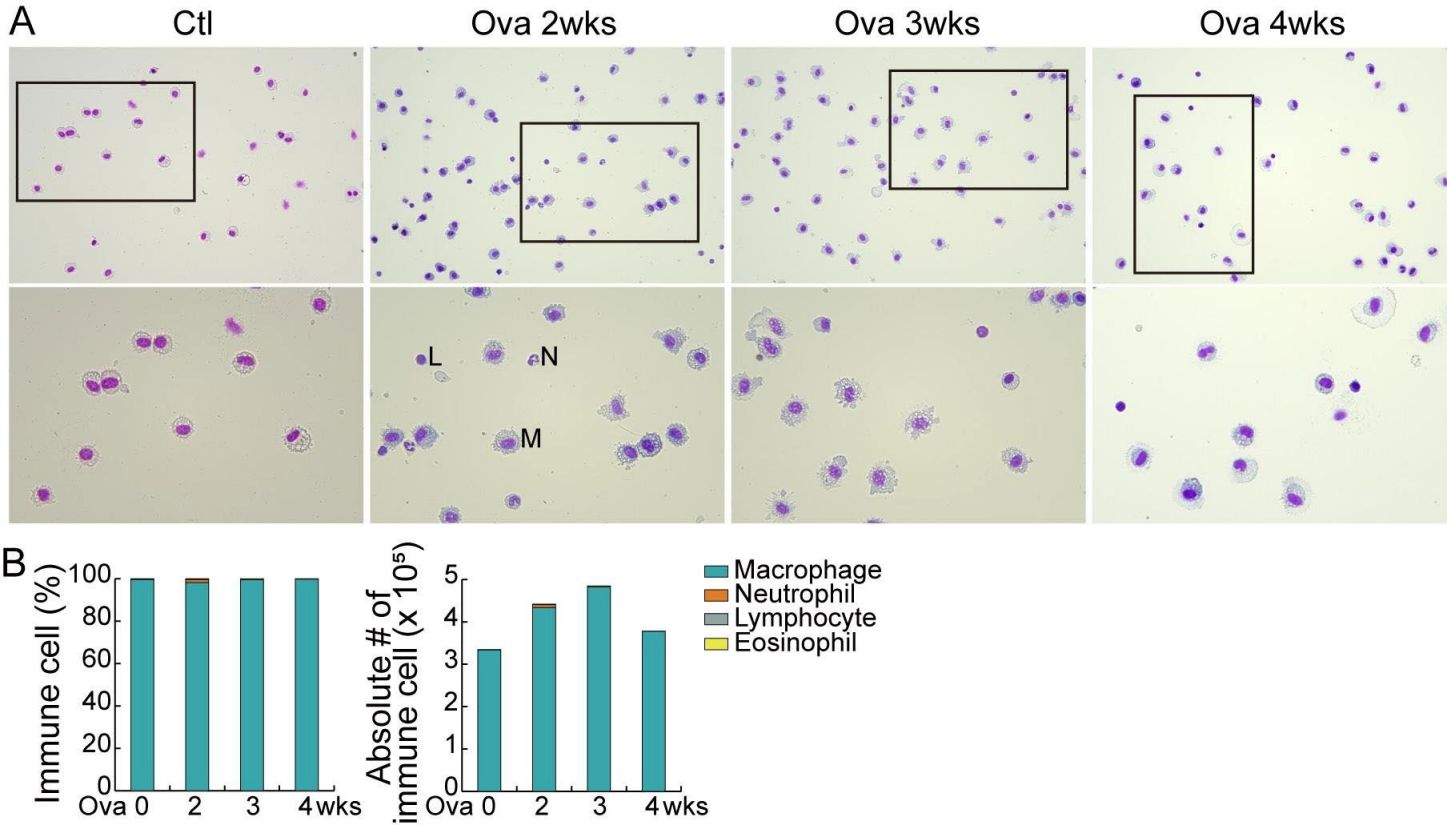
**Immune cell population in bronchoalveolar lavage fluid** Minipigs were treated with Ova during 2, 3, 4 weeks. **(A-B)** Differential immune cell count in bronchoalveolar lavage fluid (BALF) at day 29. **(A)** Microscopic images. M: macrophage; L: lymphocyte; N: neutrophil. **(B)** Number of immune cells

The bronchoalveolar lavage fluid was analyzed to determine the correlation between local and systemic inflammations. Alveolar macrophages increased until 3 weeks after Ova induction (Fig. 4 A–4B). This result is consistent with the hematology finding that monocytes increased until 2 weeks after the Ova induction (Fig. 1 A–1B). In this study, we discovered that age/body weight affects the thickness of the skin layer and Ova penetration, and it determines the success of AD porcine modeling.

## Discussion

In this study, we discovered that age and body weight are the key factors that should be considered when developing an Ova-induced AD minipig model. The skin itself is a mechanical barrier, preventing the penetration of exogenous materials. The stratum corneum, an outer layer of the epidermis [25], and tight junctions comprising claudin, occludin, and ZO-1 in the interfollicular epidermis [26, 27] form the mechanical barrier and maintain skin homeostasis. Skin diseases, such as AD, impair the skin barrier and lead to allergen penetration. To establish the porcine AD model, dermal sensitization of 2,4-dinitrofluorobenzene (DNFB), a hapten, was used [28]. However, in our previous study, we discovered that DNFB severely damaged the skin barrier due to solvent (acetone:DMSO:olive oil mixture) [24]. Thus, the Ova-induced AD is a more reliable model in terms of AD mechanisms than the DNFB-induced AD model. We established a novel Ova-induced AD minipig model by applying Ova for 2 weeks [24]. However, AD is a chronic disease; thus, the duration of the immune response should be considered. In this study, we extended the Ova sensitization until 4 weeks and performed further analyses. The results showed that 4-week Ova induction did not change the skin phenotype (Fig. 1 C), serum cytokines (Fig. 1D and E), epidermal thickness (Fig. 2 A–2B), and epidermal integrity (Fig. 2D and E), indicating that the AD minipig modeling failed regardless of a slight increase of neutrophils in the blood (Fig. 1 A–1B). Considering these unexpected results, we dissected the reason why the AD modeling failed.

The successful and failed AD models only differed in age and body weight. The successful minipig AD modeling utilized minipigs 8–10 months old and weighing 18.97 kg (Fig. 3), whereas the failed minipig AD modeling utilized minipigs 12 months old and weighing 30 kg (Fig. 2) to induce the AD. In fact, the age of the minipig affects the skin layer thickness, especially the dermis. The stratum corneum is approximately 10-µm thick, whereas the epidermis is approximately 50–64-µm thick in Göttingen minipigs 1.5–6 months old [29]. The dermis layer thickness increases from 1.15 to 2.28 mm as the minipig ages from 1.5 to 6 months [23]. The different dermal thickness also affects drug permeability and flux variation [23]. Considering these studies, we assume that low Ova penetration due to the thick dermis is related to the AD modeling failure. Most of the skin immune cells are distributed in the dermis [30]; hence, low Ova permeation might lead to a weak immune response. The results indicated that the 30-kg minipigs had a two-fold thicker dermal layer than the 18.97-kg minipigs (Figs. 2 and 3 C).

Another key factor affecting the establishment of a successful AD model is the sensitization skin site. A previous study discovered a significant regional difference in thickness among 11 different anatomic skin sites in porcine 3–4 months old [31]. The epidermal thickness ranges from 51.7 (lower jaw) to 91.7 μm (hind legs), depending on the site. The dermis has the thinnest depth at the outer ear and the thickest depth at the caudal back. Epidermal Langerhans cells are abundant at the mid-belly and inner ear skin. The thickness of the skin layer affects drug absorption, and immune cell distribution affects the immune reaction. Therefore, the same skin location should be used when sensitizing the skin. In this study, age/body weight strongly correlated with dermal thickness and Ova permeability. Although we failed to establish a chronic AC minipig model, we proved that age and body weight are critical factors when establishing an AD animal model.

## Conclusion

Age/body weight and sensitization site should be prudently considered when developing an Ova-induced AD minipig model.

## Methods

### Animals, husbandry, and feeding

Four specific pathogen free Yucatan minipigs (*Sus scrofa*) aged 12 months and weighing 25.37–30.74 kg (median weight 30 kg) were supplied by Optipharm (Osong, South Korea). They were transported in filter boxes and acclimatized for 7 days in the minipig facility at the Korea Institute of Toxicology. The experimental and control animals were housed individually in a perforated-bottom cage (850 × 895 × 845 mm) without bedding. Room temperature and humidity were regulated at 19℃–27℃ and 30–70%, respectively. Fluorescent lighting of 300–700 lx and air changes of 10–20 times/h were maintained. Water was provided *ad libitum*, and feed (PurinaMills, Gray Summit, MO, USA) was provided at the rate of 2% of the body weight per day.

## Experimental procedure

The experiment was performed from July to August 2021. To establish the AD minipig model, back skin was shaved with clippers and sterilized with 70% isopropyl alcohol, and Ova was applied using Tegaderm (cat no. 3584; 3 M, St. Paul, MN, USA). For Ova treatment, three minipigs were sensitized with 1 mg of Ova dissolved in normal saline for 2, 3, or 4 weeks. The remaining minipig served as control. For gross observation, macroscopic images were acquired on days 1, 8, 15, 22, and 29. At the end point, the minipigs were euthanized by administering pentobarbital sodium (100 mg/kg, IV; JW Pharmaceutical, Seoul, South Korea). Skin tissue samples were obtained from all six quadrants of the treated area by using a 5 mm biopsy punch for histological analysis and blood samples (10 mL) were obtained from the jugular vein for serum cytokine analysis. All the animal experiments were conducted under the Institutional Animal Care and Use Committee guideline of the Korea Institute of Toxicology (IACUC approval # 2109-0002).

## Hematology

For hematology, blood was collected into EDTA-2 K tubes, and hematology parameters were measured using an ADVIA2120i hematology analyzer (Siemens, USA).

## Enzyme-linked immunosorbent assay (ELISA)

Whole blood was collected into a conical tube and allowed to clot for 30 min at room temperature to analyze the cytokines in serum. Then, it was centrifuged for 10 min at 3000 rpm, and the supernatant was collected as serum. Porcine interleukin 13 (IL-13) and immunoglobulin E (IgE) were measured using ELISA (cat. no. ESIL13 for IL-13; Invitrogen; and NBP275006, Novus, CO, USA for IgE) in accordance with the manufacturer’s protocols.

## Masson’s trichrome staining

Masson’s trichrome staining was performed following the manufacturer’s protocol (cat no. IFU-2; ScyTek, Logan, UT, USA). Deparaffinized slides were incubated with Weigert’s iron hematoxylin, solutions of Biebrich scarlet-acid fuchsin, phosphomolybdic-phosphotungstic acid, and aniline blue. Then, the slides were rinsed with 1% acetic acid solution. The collagen connective tissues were stained blue, the nuclei were stained dark red/purple, and the cytoplasm was stained red/pink. The samples were mounted and photographed by microscopy (Zeiss; AxioVision).

## Immunohistochemistry (IHC)

The skin tissues were fixed in 10% neutral buffered formalin for overnight and then embedded in paraffin. Then, the tissue samples were sectioned (5 µm), deparaffinized, processed for antigen retrieval, blocked, and finally incubated with primary antibody, and peroxidase-conjugated secondary antibody. For peroxidase-conjugated secondary antibody, 3,3’-diaminobenzidine (DAB) substrate was used followed by hematoxylin for nuclear counterstaining. Primary antibodies against E-cadherin (Cell signaling #3195; 1:100 dilution) and claudin-1 (Invitrogen #51-9000; 1:100 dilution) were used. The samples were mounted and photographed by microscopy (Zeiss; AxioVision).

## Immune cells in bronchoalveolar lavage fluid (BALF)

Right lobes of lung were filled with PBS (total volume 500 mL) through trachea and BALF was retrieved. To determine the differential immune cell number, BALF was centrifuged (200 g, 4 °C, 10 min) onto slideglass using a Cytospin (Thermo). Then, slideglass was stained with the Diff-Quik® reagent (Sysmex Corporation, Kobe, Japan) and photographed with microscopy (Zeiss; AxioVision).

## Statistical analyses

Student’s *t*-test was used to compare two groups. Statistical significance was considered at p < 0.05.

## Electronic supplementary material

Below is the link to the electronic supplementary material.

Supplementary Material 1

## Data Availability

All data are included in Results.

## Abbreviations

AD: atopic dermatitis
Ova: ovalbumin
