# Supplementary material for: Key factors to establish the ovalbumin-induced atopic dermatitis minipig model: age and body weight
Source: Lab Anim Res. 2022 Oct 20;38:32. doi: 10.1186/s42826-022-00141-4 (PMC9585725; doi:10.1186/s42826-022-00141-4)
Supplement: Supplementary file 1 — Supplementary Material 1 [file 42826_2022_141_MOESM1_ESM.docx]

August 10, 2022

Hanna Suh, D.V.M., Ph.D.

Senior researcher

Animal model research group,

Korea Institute of Toxicology,

Jeougup, Jeollabuk-do, 56212,

Republic of Korea

+82-63-570-8525 (office)

hanna.suh@kitox.re.kr

Editor Dr. Sungsoon Fang

*Laboratory Animal Research*

Dear Dr. Fang,

First of all, I am grateful for your consideration and handling our manuscript (LARJ-D-22-00048). I am submitting the revised manuscript, **“Key factors to establish the ovalbumin-induced atopic dermatitis minipig model: age and body weight”** to be considered for publication as a case study in *Laboratory Animal Research*.

As shown in the ‘Responses to the reviewer’, we addressed all the answer against referee’s queries. In addition, to provide the accurate meaning and significance of our findings, the whole manuscript has been reorganized and edited (underlined red text).

I firmly believe that our finding will be of great interest to the readers of *Laboratory Animal Research.*

I appreciate for your consideration.

Best regards,

Hanna Suh

ORCID 0000-0002-7647-7249
